# Stairway to heaven via the highway to hell: a qualitative study on patients’ experience of knee joint replacement surgery

**DOI:** 10.1186/s13018-025-05989-5

**Published:** 2025-06-05

**Authors:** Anna Cronström, Thérése Jönsson, Gunilla Limbäck, Marcus Ljung, Caroline Ståhl, Elin Östlind

**Affiliations:** 1 Department of Health Sciences, Faculty of Medicine, Lund University, Lund, Sweden; 2 Department of ortopedics, Skåne University Hospital, Lund, Sweden; 3 Institute of Clinical Sciences at Sahlgrenska Academy, department of Orthopaedics, University of Gothenburg, Gothenburg, Sweden; 4 Department of Occupational and Physical Therapy, Sahlgrenska University Hospital, Gothenburg, Sweden; 5 Unit of Physiotherapy, Department of Health, Medicine and Caring Sciences, Linköping University, Linköping, Sweden; 6 Rehabilitation unit, Department of Orthopedic, Vrinnevi Hospital, Norrköping, Sweden; 7 Department of Orthopedic, Ängelholm Hospital, Ängelholm, Sweden

**Keywords:** Knee osteoarthritis, Knee replacement surgery, Lived experience, Qualitative research, Quality of care

## Abstract

**Background:**

Knee replacement (KR) is the most common osteoarthritis (OA) related surgery. Studies suggest that there are major international and national disparities in pre-operative information, support and access to rehabilitation which have a substantial impact on patients’ perceived outcomes of the KR. The aim of this qualitative study was to explore experiences and perceptions of the care pathway in patients who have undergone KR and subsequent rehabilitation in Sweden.

**Methods:**

Four focus group discussions were performed including in total 25 patients (16 women), median age 67.5 (range) (46–81 years), 1 to 15 months after KR. The discussion recordings were transcribed verbatim and were analysed qualitatively using content analysis with an inductive approach.

**Results:**

The analysis resulted in four categories: (1) The crooked road towards surgery, (2) Needing support throughout the whole journey, (3) Feelings of psychological distress and (4) A balancing act towards a new life. A lack of pre-operative information regarding expected pain, need of support and mental well-being were described. Although the journey was sometimes tough, patients’ expectations were, however, often fulfilled and they were in general satisfied with the decision to undergo surgery.

**Conclusions:**

Some of the challenges identified in this study may be alleviated by sufficient pre-operative information covering realistic expectations on surgery outcomes and mental aspects as well as emphatic and holistic support by healthcare providers. The result of this study will aid in the development and implementation of a national clinical practice guideline to ensure patient-centered care throughout the KR care pathway.

**Supplementary Information:**

The online version contains supplementary material available at 10.1186/s13018-025-05989-5.

## Background

Osteoarthritis (OA) is among the most common joint diseases and the fastest-growing cause of disability worldwide [1–3]. For knee OA alone, as many as 650 million people over 40 years of age lived with knee OA in 2020 [4]. Common risk factors for knee OA include older age, female sex, overweight/obesity and previous knee injury [4, 5]. First-line treatment for hip and knee OA is education, exercise, and if needed weight reduction with walking aids and pain medication as a second step [6, 7]. If patients continue to experience unacceptable pain or functional limitations after having received first-line treatment, joint replacement may be considered as an effective treatment in late-stage OA [8]. Knee joint replacement (KR) is the most common joint replacement surgery with an expected increase in the coming decades as the prevalence of OA increases, mainly due to an ageing global population, increased obesity prevalence, sedentary life styles and an increased incidence of knee injuries [2, 9]. In Sweden, a fast-track surgery program is implemented in KR, i.e., a care concept with a shortened perioperative stay that aims to improve recovery after surgery [10]. The post-operative rehabilitation is commonly conducted at a primary care clinic or at home with supervision from a physiotherapist (PT) in primary healthcare [10].

Most patients are satisfied with the long-term outcomes of the KR [11–13], but the rehabilitation may be lengthy and is frequently associated with pain, difficulty in regaining knee mobility, and difficulties in returning to work [14–16]. Previous research on patients’ experiences of undergoing KR indicates a need for support through the whole care process as well as better pre-operative information [14, 17–20]. Indeed, a positive experience of the care process seems to be related to better outcomes of the KR whereas those who lacked information and support were more prone to anxiety and worrying [17, 18]. Major disparities in information received, perceived pain and recovery process, rehabilitation plans and the overall experience of the surgery care process were also described after KR [15, 18, 20], highlighting the current variability in care of these patients and the need for relevant and high-quality clinical practice guidelines.

Considering the variability in care of these patients and the high prevalence of KR, a process of developing a person-centered and integrated clinical pathway was initiated in 2023 within Sweden’s national framework for knowledge-driven healthcare management [21]. An interprofessional working group was appointed to lead the development process, with the objective of presenting a recommended clinical pathway in 2025. A lack of up-to-date knowledge regarding patients’ experiences and perceptions of KR and the subsequent rehabilitation in Sweden was identified by the working group. In person-centered care, it is crucial to gather knowledge about patients’ experiences throughout the care pathway, and addressing knowledge gaps can lead to improved care for patients. Hence, the aim of this qualitative study was to explore experiences and perceptions of the care pathway in patients who have undergone KR and subsequent rehabilitation.

## Methods

### Design

A qualitative study design including focus group discussions was used. The Consolidated criteria for reporting qualitative research (COREQ) guided the reporting of the study [22].

### Setting

In Sweden, first-line treatment and management of OA patients and post-operative rehabilitation after KR, is carried out within primary care. Health care is primarily financed through public taxes, with a maximum total patient fee during a twelve-month period of approximately 120 USD for outpatient’s visits for all patients, aiming to minimize the financial barriers for seeking health care. There is a universal public health care guarantee which stipulates that there should be no more than three months waiting time to see an orthopaedic surgeon for assessment of KR, and there should be no more than three months waiting time for surgery after the surgery decision. Otherwise, patients have the right to be referred to another health care provider without any associated extra costs.

### Participants and recruitment

Individuals who had received a KR due to OA at health-care providers in southern Sweden were eligible to participate in the study. Inclusion criteria were: >1 month and ≤ 18 months post-surgery at the time for the focus group discussion. Exclusion criteria were: having rheumatoid diseases, cognitive impairment, comorbidities significantly affecting rehabilitation (e.g., neurological disease, chronic obstructive pulmonary disease) or not being able to understand or speak Swedish.

A Lund University webpage with information about the study was published online. A link to the website was published in Facebook groups for people with OA, on the website of the Swedish Rheumatism Association, and on Lund University’s website dedicated to osteoarthritis. The link was also disseminated through social media posts and shared with physiotherapy colleagues in southern Sweden.

The Principal Investigator of this study (EÖ) (PT) screened all participants according to inclusion/exclusion criteria. We used a purposive sampling method aiming to include individuals of both sexes, with a spread in age and time since KR both with and without previous joint replacements. The included participants then signed up for the discussion group that was held at their nearest location.

### Data collection

Four focus group discussions were conducted at three different locations in southern Sweden with between five and seven participants in each group. All discussions were moderated by EÖ (PhD) with assistance by the co-authors TJ (PhD) (discussion one and two), CS (BSc) (discussion three) and AC (PhD) (discussion four). EÖ, TJ, CS and AC are female, registered PTs with experience from post-operative rehabilitation and OA research. EÖ and AC have experience in qualitative research. None of the authors that were present during the discussions had any previous relation with the participants. Further information about the study was provided when they registered to participate in the study. The participants completed a brief questionnaire with background questions (i.e., age, sex, type of KR, time since KR, any previous joint replacements) and signed the informed consent form at the time of the discussion.

The group discussions followed a consistent format. When arriving, participants were offered beverages and the opportunity to meet and interact with each other. The moderator began with a brief introduction, highlighting the importance of open and free communication. It was stressed that there were no ‘right’, or ‘wrong’ responses and participants should feel comfortable sharing their experiences. Additionally, participants were requested to keep the discussion content confidential. The discussion that followed was audio-recorded and conducted according to a structured questioning route, which included an opening question, introductory questions, key questions, and concluding questions (Appendix 1) [23]. The questioning route was pre-designed by the author group together with a patient partner [24] from the Swedish Rheumatism Association and used consistently across all four focus group sessions. The questions were primarily open-ended and crafted to address the study’s objectives. Participants were encouraged to engage in discussions with each other. Follow-up questions or questions directed at specific participants were posed as necessary. The assistant took field notes throughout the sessions. At the conclusion of each session, the assistant provided a verbal summary of the discussion, inviting participants to offer comments or corrections. Following each focus group, the moderator and assistant held a short debrief to reflect on the discussion’s content. The focus group discussions lasted between 89 and 106 min and were conducted at a primary clinic (*n* = 1), at a regional hospital (*n* = 1) and at University facilities (*n* = 2) in April-May 2024.

### Data analysis

The group discussions were transcribed verbatim by EÖ, ML and a part-time student employee.

Qualitative content analysis with an inductive approach (no prespecified framework or theory was used) according to Elo and Kyngäs was applied [25]. The transcribed group discussions were seen as unit of analysis. The transcribed discussions were analyzed by two of the authors (EÖ and AC). First, the transcripts were read through several times to become familiar with the data. Meaning units relevant to the study aim were identified, condensed, and coded. Codes were grouped in sub-categories and categories based on the meaning of the codes. Both authors then reread the original transcripts for validating purposes and to ensure that no relevant aspects were missed. Each step was then discussed and triangulated with a third author (GL) (PhD), who have experience in qualitative research, until final agreement on categories and subcategories was reached. Selected key quotes were highlighted. Microsoft Excel and the software program NVivo 14 (QSR International Pty. Ltd) were used in the analysis process.

## Results

Twenty-five persons who had undergone KR the last 15 months participated in this study. Median age was 67.5 years and a majority (64%) were women (Table 1).

**Table 1:** Participant characteristics

	(*n* = 25)
**Age (years)**,** median (min-max)**	67.5 (46–81)
**Sex**,** (n)**	
Female	16
Male	9
**Months post-op**,** mean (min-max)**	6 (1–15)
**Type of replacement**,** (n)**	
Total knee replacement	21
Uni-compartmental replacement	4
**Have previous joint replacements**,** (n)**	
Yes	15

SD standard deviation

### Categories with subcategories

The focus group interviews resulted in four categories with associated subcategories: (1) The crooked road towards surgery with three subcategories, (2) Needing support throughout the whole journey with two subcategories, (3) Feelings of psychological distress with three subcategories and (4) A balancing act towards a new life with three subcategories (Table 2). Examples of the analysis process can be found in Table 3.

**Table 2:** Categories with associated subcategories

Category	Subcategory
**The crooked road towards surgery**	Longstanding severe symptoms
	Varying access to surgery
	Disparities in preparatory information
**Needing support throughout the whole journey**	The importance of empathetic support from healthcare
	Feelings of helplessness after surgery
**Feelings of psychological distress**	Unprepared for psychological distress
	Feeling a lack of holistic perspective
	Continued worry
**A balancing act towards a new life**	Load optimization is challenging
	Factors influencing rehabilitation progress
	Acceptance and readjustments

**Table 3:** Examples of the analysis process

Meaning unit	Code	Subcategory	Category
*“I have to say that they made it feel so warm and safe. Even though you are scared…”*	Well taken care of	The importance of empathetic support from healthcare	Needing support throughout the whole journey
*“What I want to highlight now is something no one has brought up—the issue of mental health. I felt so*,* so bad// but no one told me”*	No information on the mental part	Unprepared for psychological distress	Feelings of psychological distress
*“I received sufficient information before both of my surgeries”*	Well informed	Disparities in preparatory information	The crooked road towards surgery
*“I think that acceptance is part of this change. If you have thought that you need to do certain things*,* you might be forced to rethink it//so it’s part of my personal process*,* I think*	Accepting their new situation	Acceptance and readjustments	A balancing act towards a new life

### The crooked road towards surgery

This category includes three subcategories, a) *Longstanding severe symptoms*, b) *Varying access to surgery* and c*) Disparities in preparatory information*.

#### Longstanding severe symptoms

Most participants described that they had longstanding symptoms related to their knee OA prior to their surgery although experiences of a more sudden onset of disabling OA were also mentioned. The knee OA had a major impact on their daily activities and quality of life. They experienced severe pain and reduced function that prevented them from sleeping and doing everyday tasks, such as walking, working and exercising. This also affected people close to them, which contributed to the decision regarding surgery.

*“I couldn’t take it anymore. It really affected my life situation. It was like ups and downs*,* but then things got really bad for a long period. I just felt like– I can’t even walk 2 km without being in so much pain.”* (FG4).

#### Varying access to surgery

The participants expressed disparities regarding access to surgery. While some participants perceived that it was easy to get an assessment from an orthopedic surgeon and the subsequent KR surgery, others described that they had to wait a long time for surgery, despite severe symptoms. The care pathway from primary healthcare to surgery held several bureaucratic obstacles. They perceived that they had been directed back and forth between different healthcare providers and that they had to put a lot of pressure on healthcare professionals to get any help at all. Having comorbidities was an important factor that limited the number of clinics that could conduct the KR.

*“I know that there are people who have to fight for a long time before they are allowed to have this surgery*,* but I didn’t have that experience either time. For that*,* I’m grateful.”* (FG4).

*“I feel that this process took a very*,* very long time. I had problems for many years and didn’t really understand what the issue was. But when I started to realize it and began pushing my primary care physician to investigate it*,* it took a very*,* very long time before I was heard. She* [the primary care physician] *didn’t think it was the right time for that.”* (FG2).

#### Disparities in preparatory information

Some participants were satisfied with the information they received prior to the surgery and felt well prepared for both the surgery and for the postoperative rehabilitation period.

*“I received sufficient information before both of my surgeries. I was provided with detailed instructions on how to prepare physically*,* what happens during the surgery*,* information about medications*,* and much more”* (FG4).

However, several participants expressed that they had received too little information prior to the surgery. They were unprepared for the pain experienced after the surgery, and they lacked information on what kind of assistive devices they would need to facilitate their everyday life and their need for assistance from family or friends. They said that they had to look up all the information by themselves on the internet and wished that there was someone they could have called to ask questions. They also expressed that it was remarkable that the pre-operative information could be so different for the same treatment.

*“To receive some information about expected pain… I had never imagined it would be so difficult. The first 14 days were really tough—I stayed in a separate room with an extra bed to not disturb my wife. I was up walking*,* then sitting again*,* almost screaming in pain. I took pills and didn’t sleep much. I hadn’t expected that.”* (FG4).

### Needing support throughout the whole journey

This category includes two subcategories (a) *The importance of empathetic support from healthcare* and (b) *Feelings of helplessness after surgery*.

#### The importance of empathetic support from healthcare

All participants emphasized the importance of an empathetic approach by the healthcare providers. However, the participants had varying experiences in this regard. A few participants perceived that they were badly treated and almost harassed. They had experiences of not being listened to, not receiving person-centered care and compared the hospital to a factory with an assembly line. In contrast, many participants described a warm and welcoming care environment with supporting healthcare providers. The kind treatment made them feel very safe and helped them overcome the fear of the surgery. Some of the participants described that either the surgeon or a nurse had called them during the first weeks after surgery. They could also call the orthopedic clinic day and night. This made them feel safe and helped them to handle their pain.

*“She was so rude [nurse]*,* and it’s a bit sad when all the other staff were super nice. And then in the recovery room*,* she was running around harassing me and making snide remarks about this and that*,* so I reported it to the clinic manager.”* (FG3).

*“I have to say that they made it feel so warm and safe. Even though you are scared*,* even though you are in pain*,* and even though you need a lot of help*,* you feel safe*,* and that means a lot”* (FG1).

The PTs in primary care were also described as very important support during the rehabilitation. A majority perceived that the PTs provided valuable information and explanations for their physical complaints and guided them in finding a balance in physical activities and training.

*When I did the exercises there with her [the PT]*,* I felt a bit happier*,* and she said*,* ‘Yes*,* you can continue. We can challenge your knee a bit more because you are ready*,*’ and then I got excited about life again*,* so to speak”* (FG1).

Some participants did, however, discontinue rehabilitation at an early stage due to a perceived lack of support and engagement from their PTs.

#### Feelings of helplessness after surgery

The participants were used to taking care of themselves prior to the surgery, but afterwards they had to rely on others for help with almost everything. They felt that they were unprepared for how much help they would need after their surgery due to their pain. Several participants noted that they couldn’t do the simplest things, like go to the toilet without support. Feelings of being prisoners in their own homes for a period after the surgery emerged. They were dependent on someone to help them, which evoked feelings of helplessness and to be a burden for their families and friends.

*“I mean*,* they just assume you have someone at home to help you. Yes*,* I’m married*,* yes. But*,* well*,* he works*,* right. So*,* I’m just at home. I couldn’t manage on my own. I couldn’t do anything. I could barely even get to the toilet*,* and I was like*,* ‘Wait*,* I can’t do this. I can’t.’ And then there was so much pain*,* and when it was time to eat*,* I needed so much more help than I was prepared for*,* because I was in so much pain.”* (FG1).

Talking to people that had been through the same journey was, however, expressed as very valuable. Some participants described that they made friends with people that also had a KR at the same time or that they were part of groups on social media where they could share common experiences. Having this peer support helped them to overcome challenges during the rehabilitation and regain their confidence. However, they wished that the healthcare had arranged group meetings after KR surgery where they could meet people in the same situation and share experiences.

*“You can tell what you have done and share your experiences… and*,* like now*,* we have met a few people*,* and you get to hear that you’re not alone in what you’re going through. There are more people in the same situation*,* and that is a big advantage*,* actually”* (FG4).

### Feelings of psychological distress

This category includes three subcategories a*) Unprepared for psychological distress*, (b) *Feeling a lack of holistic perspective* and (c) *Continued worry.*

#### Unprepared for psychological distress

The participants described that they were unprepared for how the decision on surgery and the surgery itself would affect their mental wellbeing. They expressed feelings of considerable worry prior to the surgery, with one participant reporting severe mental distress that required counseling to manage the emotional burden during this period. Many participants experienced the psychological distress after the surgery to be more challenging than the physical symptoms. They perceived that the pre-operative information focused only on practical matters and physical symptoms. No one had informed them about the potential psychological impact of the surgery or that depression and similar symptoms are common after surgery.

*“When you have this four-hour information meeting one month before*,* it would have been interesting if there had been someone there who could explain what might happen afterwards. Especially about feeling low and stuff*,* because you mostly get information about what happens up until you’re operated on and ready to go home. How everything works—it’s very detailed*,* all the information you need—but afterwards*,* you’re kind of left without anything. And then*,* you read about some people who are completely devastated*,* sitting and crying*,* and some who laugh and are happy—it’s so different how people react”* (FG3).

#### Feeling a lack of holistic perspective

The general perception among the participants was that the healthcare providers failed to see the whole person, which caused dissatisfaction. The only focus was on the surgery or the knee itself and this fragmented approach left patients feeling unsupported, especially regarding the psychological and emotional aspects of the care process. Several participants highlighted the need for a comprehensive perspective that encompasses physical, mental, and emotional health before, during, and after surgery.

*“It’s not just all the stuff with the leg*,* it’s maybe the mental part too that’s important*,* and I probably missed that [from the healthcare] when I think about it now.”* (FG3).

#### Continued worry

The participants described that they were still worried about their knee long after the surgery. It took substantial time before the prosthesis felt like their own knee and, in the beginning, they did not recognize their knee. They did not trust the knee to function properly and were often afraid of kneeling or doing something that they thought would be unpleasant or would damage the prothesis. They therefore avoided activities and took fewer risks to minimize the risk of having to go through a new surgery.

*“I’m avoiding everything that could stress the prosthesis to reduce the risk of having to replace it in ten years*,* and I’m terrified. So*,* I bought a tricycle to minimize the risk.”* (FG2).

### A balancing act towards a new life

This category includes three subcategories (a) *Load optimization is challenging*, (b) *Factors influencing rehabilitation progress and* (c) *Acceptance and readjustments*.

#### Load optimization is challenging

There was consistency in the focus groups regarding their perceptions of difficulties in finding a balance between activities and rest. When they felt better it was very easy to do activities that eventually overloaded the knee. It was seen as a Catch-22 situation, they experienced an immediate setback just when they felt a little better and increased the loading. Some participants also described feelings of anxiety if they hadn’t performed the exercises often enough.

*“In the end*,* it became too much—the knee swelled*,* and it started hurting. Then I was told that I had to calm down. But it’s not easy to do that when you’re not in pain*. (FG4)

*“If I hadn’t done the exercises that day*,* I got this anxiety—I need to do this tonight*,* I must*,* and then I get anxiety about finding the balance*.” (FG2).

#### Factors influencing the rehabilitation progress

Adhering to the exercises was described as important for progress in the rehabilitation. It was, however, perceived as challenging to balance the need for exercise with the demands of daily life. To balance the characteristics of physical and psychological demands from work and the termination of sick leave with the time they needed to invest in rehabilitation was perceived as challenging and stressful. While some participants were on sick leave up to 5 months after the surgery, others had only 3 weeks of sick leave. Going back to work was described as physically and mentally difficult. Not finding time for physiotherapy while working full-time was expressed as stressful by the participants since they knew how important it was for the rehabilitation of the knee.

“*Since my work is not that physically demanding*,* I’m supposed to be back earlier. But then you don’t have time for physiotherapy*,* and that was the most important thing.”* (FG4).

While some participants described that they had almost unlimited access to a PT during the rehabilitation period others expressed that they had only met the PT a few times and that they had to manage their rehabilitation on their own. Many participants perceived that the supervised part of their rehabilitation was too short and that it was hard to find motivation to continue performing the exercises at home.

*“It’s difficult to fit it in [exercise]*,* and life just doesn’t add up. And yes*,* I can do a lot at home*,* but it’s not the same.”* (FG4).

Simultaneously, having functional impairments or having undergone a recent KR on the other knee was also described as negative influences on the rehabilitation. Persistent symptoms and not having regained function from the first surgery made it harder to go through with the rehabilitation for the second surgery.

*“It was also much more difficult to have two new knees than it was to have one new and one old knee. And then I think it also has to do with the fact that I didn’t have*,* well*,* the strength I had built up in the first knee—it had kind of gone down. It had gone down. I wasn’t as fit before the second surgery”* (FG4).

#### Acceptance and readjustments

By undergoing a KR, the participants held expectations that they would be able to do the things they previously used to do but couldn’t do anymore due to their symptoms. This included expectations of being pain-free and sleeping properly and having a social life - just to live a normal life and do ordinary things.

After the KR, participants did, however, highlight the difficulty of reconciling their current physical limitations with the activities they once enjoyed, such as sports, walking long distances, or traveling. Many expressed a strong desire to return to previous activity levels, including ambitious goals like skiing, golfing, or running, but also acknowledged the need to adjust expectations and pace their recovery. Acceptance was seen as a gradual process tied to redefining personal goals and priorities, such as focusing on achievable activities like walking a few kilometers, gardening, or enjoying social aspects of sports. The process helped them grow as a person and to cope with their new situation.

*“I think that acceptance is part of this change. If you have thought that you need to do certain things*,* you might be forced to rethink it. I’m 66 years old today*,* so it’s part of my personal process*,* I think. What is good for my body if I want it to last*,* which I do*,* for as long as possible. So*,* I think like that. Even though I have done a lot before*,* I don’t feel at all that I need to do it today*,* and that feels good in a way. To be able to let it go.”* (FG3).

Although the journey sometimes was tough, nearly all participants were happy that they had undergone the surgery. For the participants that were at the late stage in their rehabilitation, their expectations were mostly fulfilled and sometimes exceeded. They were able to play golf, travel, sleep without pain, walk long distances, and engage in social life. The participants expressed that they had got their life back and could now do things that they hadn’t been able to do for many years. They perceived that the KR had a significant impact on their quality of life, at least after the initial rehabilitation period. A few participants were completely satisfied with the whole care process.

*“I’m really satisfied. It has gone really well. So*,* it’s great. Not having pain*,* being able to lie straight on my stomach on the sunbed—I thought about that during my last holiday*,* it was wonderful. I lay there like… well*,* it’s such an incredibly big difference*,* it’s fantastic.”* (FG3).

## Discussion

In this study, we explored patients’ experience of the KR care pathway in individuals with knee OA. The categories reflected a journey full of obstacles including unexpected pain and psychological distress with disparities in pre-operative information and need of support. However, despite the perceived challenges, the participants were in general satisfied with the decision to have surgery.

### Disparities in pre-operative information

In the current study, many patients expressed that they were completely unprepared for the level of post-operative pain and the impact this would have on their everyday life during the rehabilitation. They wished to have received more information in order to be better prepared after the KR in terms of pain medication and assistive devices needed to ease their symptoms. A systematic review has concluded that adequate pre-operative information is a prerequisite for feeling safe when undergoing a KR. Sufficient information may also aid patients in having realistic expectations about post-operative pain and the overall well-being during the subsequent rehabilitation period. Consequently, too little or contradictory information may lead to uncertainty and anxiety [26]. Nevertheless, several studies report on disparities in the information provided to the patients before the surgery [15, 18, 20, 26]. In particular, many patients lacked information on the length of the rehabilitation period and the expected level of post-operative pain [14, 17, 18, 20]. This corroborates the results of two previously published studies on patients’ experience of KR in Sweden. In 2019, Skogö Nyvang et al. [18] published a qualitative analysis on patients’ experience of KR and subsequent rehabilitation (interviews conducted 2014–2015) and Berg et al. [19] investigated patients’ experience of a fast-track program in KR surgery (interviews conducted in 2017). In both these studies, the patients highlighted a lack of information regarding expected pain and symptoms and that “no one told them how hard it would be” [18, 19]. Although there are several attempts to improve the clinical pathway for patients undergoing KR in Sweden, such as knowledge-driven healthcare management [21], the result from the current study indicates that some of the negative experiences described at 8–11 years ago, are still the same today.

### Needing support throughout the whole journey

The participants in this study had varying experiences of the support given by the healthcare providers. Some participants described that they received empathic support throughout the whole care process to help them handle their fear before the surgery and symptoms post-surgery. However, others experienced a more hostile environment that led to an overall negative experience. The PTs were perceived as important for guiding the balance between exercise and rest as well as for motivational support during the rehabilitation post-surgery. Nevertheless, disparities in access to rehabilitation and perceived support from PTs were evident among the participants. Previous research suggests that adequate pre-surgery information and support through the whole care process are highly related to better outcomes [17, 18] and that neuromuscular exercise is the most effective rehabilitation intervention for improving outcomes after KR [27]. To promote satisfaction rate after KR and ensure equal care for all patients, implementation of thorough clinical practice guidelines pre-surgery and throughout the rehabilitation, including information and support as well as access to physical therapists are, thus, highly warranted. In line with previous studies [15, 18, 19, 28], the participants in the current study described peer support to be very helpful. Hearing stories from people that have gone through the same process were often described as more valuable than information and support from healthcare. The participants mostly engaged in different groups on social media to interact with fellow individuals with KR but requested group meetings arranged by the healthcare. Organized patient support groups before and early after surgery may, thus, be one feasible way to meet patients need of support as well as promote realistic expectations of the rehabilitation process.

### Psychological distress

Another aspect that was evident among the participants in our study was the perception of the healthcare providers’ lack of a holistic perspective when interacting with the patient. The main focus of information and support given to the patient was on the surgery and functional aspects and was indeed very knee specific. The participants felt that they were not seen as a whole person and that important aspects related to mental well-being both before and after surgery were mostly overlooked. They further described that they were completely unprepared for the psychological distress that would be associated with the surgery and subsequent rehabilitation. Many participants experienced the mental part to be more difficult to deal with than the functional limitations and wished that someone had informed them how the surgery would affect their overall mental well-being. In addition, many patients had continuous worries with regards to the solidity of the prothesis and were afraid of doing anything that would cause harm and increase the risk of revision surgery. This is also reflective of the results of previous studies where patients reported anxiety and fear related to the surgery [17, 19] which further affected their ability to assimilate the surgery-related information provided to them [17]. Systematic reviews as well as qualitative studies have concluded that psychological distress, such as pain catastrophizing, anxiety and high mental distress, lead to greater post-operative and chronic pain as well as worse function after KR [29–31]. To improve postoperative outcomes in these patient, preoperative interventions targeting psychological, such as e.g., education, music therapy, pain coping skills training, imagery and biofeedback, may be helpful [32]. Given this, more comprehensive information covering also possible psychological aspects of the surgery process as well as screening for psychological distress pre-operatively may further improve outcomes after KR as well as the patients’ overall experience of the surgery.

In line with previous qualitative studies [28], many participants in the current study expressed continued worry late in the rehabilitation period. They were afraid of doing something that could damage the knee prothesis which in many cases prevented them from doing things they wanted to do. This further indicates a need for continued long-term support and follow-ups by a PT.

### A balancing act towards a new life

The journey from the surgery decision to a functional knee post-surgery was in general described as difficult with many obstacles along the road. Yet, most patients were satisfied with their decision and described that, in the end, their expectations were fulfilled and that they “had got a new life”. This may in part be attributed to readjustments and acceptance of their new situation. Randall et al., [33] have explained patient satisfaction after KR using a model of *adequacy* and *adapting*, where adequacy, i.e., a functional knee and fulfilled expectations, is highly dependent on the person’s ability to adapt. This includes processes where the patient adjusts both physically and cognitively leading up to acceptance of their new situation, potentially including revision of any pre-surgery expectations [33]. Several participants in the current study described that they had adjusted their expectations and come to peace with that the rehabilitation may take longer than they expected and that they may never be able to do some activities that they had done when they were younger. Accepting their new situation and redefining their expectations were seen as an important part of their personal development and helped them cope during the rehabilitation rollercoaster. Pre-surgery discussions of the patient’s expectations of the surgery and subsequent rehabilitation are, therefore, of utmost importance.

### Clinical implications

Person-centered care emphasizes a partnership between healthcare providers and patients to improve patient health, taking individual capabilities, needs and goals into account [34]. This partnership could be implemented on three levels (i) *direct care* where patients receive information about the diagnosis and treatment options, individual goals are discussed and treatment is ultimately based on shared decision, (ii) *organizational design and governance* and (iii) *policy making* where the patients are asked about care experiences and health-care issues and can be in advisory positions at healthcare clinics and/or be included in development and implementation of healthcare pathways and policies [35]. To improve patient care in patients undergoing KR in Sweden, the development of a national clinical pathway in collaboration with patient representatives has started [21], which is an important step towards person-centered care [36]. The current study is part of that work, and, as per definitions above, represents patient engagement at *organizational* and *policy making* levels. Importantly, the results reveal that there is room for improvement in person-centered care at the *direct care* level, where disparities in pre-operative information and access to rehabilitation were reported as well as a lack of empathetic and holistic care. The result will highlight areas in the clinical pathway that should be prioritized to improve patient-centered care.

### Strengths and limitations

All focus groups were conducted with the same main moderator ensuring consistency in the discussions across the focus groups. Neither the main moderator nor any of the co-moderators had any previous relation with the participants, which facilitated open and free discussions among the participants. The participants were purposefully selected including both sexes with a wide range of age and time since surgery with both total KRs and uni-compartmental from different care-units in south of Sweden allowing us to receive rich and varied data on the experience of KR surgery. No additional information was gained from the last two focus groups indicating saturation of the data. Throughout the data analysis, reflexivity has been considered, i.e. AC and EÖ worked separately during the data analysis and continuous discussions were held to prevent potential influences of the authors pre-understanding and previous experiences. A signature representing the specific focus groups was also reported after each quotation to increase transparency and trustworthiness (i.e., that all focus groups were represented) of our results.

This study is associated with some limitations. The participants in this study had a wide range of time since surgery (1–15 months). It is possible that the data gained from those who performed the surgery almost one and a half years earlier may be prone to recollection bias. On the other hand, participants that were in a very early rehabilitation phase may still exhibit considerable pain and could not contribute data on the entire journey and whether their expectations were fulfilled or not. This could potentially have skewed the result towards a more negative experience of the KR. This approach did, however, give us a wide variation in the data as well as facilitating true experiences from the whole surgery process. A long those lines, approximately 60% of the participants had a previous joint replacement. In the current study, those having a previous joint replacement were mostly describing worse experiences of the rehabilitation process due to persistent functional limitations from the first surgery. Although both positive and negative experiences were reported, it is also possible that patients with more negative experiences of the KR journey were more interested in participating in the study and all of the above should be considered when interpreting the results of this study. For practical reasons, we did only include participants from care units in the south of Sweden and the results may, thus, not be generalized to patients performing their KR in other care units across Sweden. Qualitative research has limited generalizability, however we consider the transferability to be relevant since the result from the current study does corroborate with the results from similar national and international studies, indicating that the geographic setting of this study had little influence on the results.

## Conclusion

Some of the challenges identified in this study may be alleviated by sufficient pre-operative information covering realistic expectations on surgery outcomes and mental aspects as well as emphatic and holistic support by healthcare providers. The result of this study will aid in the development and implementation of a national clinical practice guideline to ensure patient-centered care throughout the KR care pathway.

## Electronic supplementary material

Below is the link to the electronic supplementary material.

Supplementary Material 1

Supplementary Material 2

## Data Availability

The data used in this study (i.e., recorded focus group material) contains sensitive information about the study participants and they did not provide consent for public data sharing. The current approval by the Regional Ethical Review Board in Sweden does not include data sharing. A minimal data set could be shared by request from a qualified academic investigator for the sole purpose of replicating the present study, provided the data transfer is in agreement with EU legislation on the general data protection regulation and approval by the Swedish Ethical Review Authority.

## Abbreviations

KR: Knee replacement
OA: Osteoarthritis
COREQ: The Consolidated criteria for reporting qualitative research
PTs: Physiotherapists
SD: Standard deviation
FG: Focus groups
