# Supplementary material for: Stairway to heaven via the highway to hell: a qualitative study on patients’ experience of knee joint replacement surgery
Source: J Orthop Surg Res. 2025 Jun 5;20:570. doi: 10.1186/s13018-025-05989-5 (PMC12139267; doi:10.1186/s13018-025-05989-5)
Supplement: Supplementary file 1 — Supplementary Material 1 [file 13018_2025_5989_MOESM1_ESM.docx]

**Interview Guide;** **Structure based on Krueger & Casey (2000)**

**Opening Question – Round of Introductions (First name)**
– Have you undergone a joint replacement surgery before?

**Main Question**
– How would you describe your journey from undergoing knee replacement surgery up to where you are today?

**Follow-up Questions**
(to be asked if the discussion ends without these areas being addressed):

- What made you decide to have the surgery?
- What was your experience of the surgery and the care you received?
- How did you experience the rehabilitation process?
- What expectations did you have regarding the outcome of the surgery?
- Have your expectations been met? (Yes/No + possible follow-up question)
- What are your thoughts on a potential surgery for your other knee?

**More Specific Questions**

- What worked well and what could have been improved regarding healthcare?
- How well did the preoperative information meet your needs?
- How did the transition from the orthopedic clinic to primary care (and vice versa) work?
- How have sick leave and returning to work been managed?
- Are there any other thoughts you feel are important to adress regarding knee replacement surgery?

**Closing Questions**
The co-moderator summarizes the questions and the main themes that have emerged, and asks participants if there is anything they would like to add.
